# Fabrication of MIL-53(Al)-Incorporated Cellulose Acetate Mixed Matrix Forward Osmosis Membrane for Seawater Desalination

**DOI:** 10.3390/nano16211417

**Published:** 2026-11-04

**Authors:** Zimeng Cao, Caixia Cheng, Mengmeng Wang, Yanjiao Liu, Zhongpeng Wang, Xiuju Wang, Liguo Wang

**Affiliations:** School of Water Conservancy and Environment, University of Jinan, Jinan 250022, China

**Keywords:** desalination, forward osmosis membrane, cellulose acetate, MIL-53(Al)

## Abstract

Forward osmosis (FO) membranes have received growing attention in the field of water treatment. Nevertheless, their practical application in seawater desalination remains restricted, underscoring the imperative of developing advanced membrane modification techniques. In this study, we synthesized metal–organic framework MIL-53(Al) nanoparticles via a hydrothermal method and incorporated them into cellulose acetate (CA) membranes to fabricate a CA/MIL-53(Al) mixed-matrix FO membrane. The synthesized MIL-53(Al) nanoparticles were characterized by X-ray diffraction (XRD), Fourier transform infrared spectroscopy (FTIR), scanning electron microscopy (SEM), and N_2_ adsorption–desorption analysis. The effects of MIL-53(Al) loading, pore-forming agent content, mixing temperature, and coagulation bath temperature on the membrane performance were systematically investigated to optimize the fabrication conditions. Under the optimal conditions, the prepared CA/MIL-53(Al) mixed matrix FO membrane exhibited a water flux of 65.71 L/(m^2^·h) and a reverse salt flux of 2.20 g/(m^2^·h). Compared with the pristine CA FO membrane, the incorporation of MIL-53(Al) significantly improved the membrane permeability and separation performance. These enhancements were attributed to the improved hydrophilicity and optimized membrane structure induced by MIL-53(Al) nanoparticles. This study demonstrates that MIL-53(Al) is an effective nanofiller for the fabrication of high-performance CA-based FO membranes and provides a promising strategy for seawater desalination applications.

## 1. Introduction

FO has emerged as an attractive membrane separation technology due to its advantages of low energy consumption, reduced membrane fouling, and high solute rejection capability [1,2]. It has been widely applied in various fields, including seawater desalination [3,4], wastewater treatment [5,6,7], food processing [8], beverage concentration [9,10], bioproduct manufacturing [11], and pharmaceutical production [12,13]. As the core component of FO processes, FO membranes play a decisive role in determining separation efficiency. An ideal FO membrane should simultaneously exhibit high water permeability, excellent salt rejection, low reverse salt flux, strong hydrophilicity, structural stability, and antifouling properties [14,15].

CA has been extensively investigated as one of the most widely used materials for FO membrane fabrication owing to its intrinsic hydrophilicity, low fouling tendency, cost-effectiveness, and good biocompatibility [16]. However, conventional CA-based FO membranes still suffer from several limitations, including relatively low water flux, severe internal concentration polarization (ICP) [17], difficulty in precisely controlling pore structures, and insufficient long-term stability [18]. These drawbacks restrict their practical application in demanding processes such as seawater desalination [19]. Therefore, developing effective modification strategies to simultaneously enhance water permeability and salt rejection performance remains a critical challenge for CA-based FO membranes. Recent progress in chemically integrated nanomaterial–polymer hybrid membranes for water purification has shown that interfacial design and transport mechanisms are central to addressing this challenge; nanofiller–polymer interactions can regulate pore structure, polymer chain packing, and water transport pathways, thereby decoupling water transport from pore enlargement and enabling simultaneous improvements in permeance and selectivity [20].

Recently, metal–organic frameworks (MOFs) have attracted extensive attention as a class of crystalline porous materials due to their high porosity, large specific surface areas, and tunable pore structures [21]. Constructed by the coordination of metal nodes/clusters with organic ligands [22], MOFs possess diverse architectures and adjustable physicochemical properties. As inorganic–organic hybrid materials, MOFs generally exhibit favorable compatibility with polymer matrices [23,24,25]. Consequently, MOFs have been widely explored in various applications, including storage [26,27,28], separation [29,30,31], and catalysis [32,33,34]. For example, Cu–NH_2_-MIL-125(Ti) has been incorporated into a PVDF matrix to fabricate photocatalytic composite membranes for high and stable hydrogen generation, where the PVDF matrix protected the moisture-sensitive MOF and suppressed particle aggregation [35]. More importantly, their unique structural characteristics and functional versatility provide significant opportunities for regulating membrane microstructures and improving separation performance.

The Materials of Institut Lavoisier (MIL) series represents an important class of MOFs constructed from trivalent metal ions (e.g., Al^3+^, Fe^3+^, and Cr^3+^) coordinated to terephthalic or trimesic acid ligands. Among these materials, MIL-53(Al), consisting of Al^3+^ centers and terephthalate linkers, has attracted particular interest for its large surface area, excellent thermal stability, and robust framework structure [36]. The strong coordination between aluminum centers and oxygen atoms from carboxylate groups endows MIL-53(Al) with outstanding stability in aqueous environments [37]. Therefore, incorporating MIL-53(Al) into polymer matrices is an effective approach to extend its application in advanced membrane separation technologies [38].

Chen et al., ref. [39], prepared multifunctional MIL-53(Al) derivatives through a microwave-assisted solvothermal method and incorporated them into a poly(ether-block-amide) (PEBA) matrix to fabricate MIL-53(Al)-X/PEBA mixed matrix membranes. The incorporation of MIL-53(Al) improved membrane water stability through the robust Al–O framework, while functional groups on the ligands enhanced membrane hydrophilicity and interfacial compatibility, resulting in improved pervaporation performance for n-butanol wastewater treatment. Tian et al., ref. [38], fabricated dense MIL-53(Al) inorganic membranes via an in situ conversion strategy using γ-Al_2_O_3_ as an intermediate layer. The strong coordination framework of MIL-53(Al) provided excellent acid and water stability, while its intrinsic hydrophilic channels promoted selective water transport. Meanwhile, chemical bonding between the membrane layer and substrate enhanced mechanical stability, enabling efficient dehydration of highly concentrated acetic acid wastewater. Wei et al., ref. [40], synthesized a La-doped MIL-53(Al)/ZnO composite through a combined hydrothermal and calcination process. The obtained composite exhibited enhanced photocatalytic degradation efficiency toward sulfamethazine, demonstrating potential for environmentally friendly wastewater remediation.

Based on these considerations, this study aims to enhance the permeability and salt rejection performance of CA-based FO membranes through MIL-53(Al) incorporation. MIL-53(Al) nanoparticles were synthesized via a hydrothermal method and characterized by X-ray diffraction (XRD), Fourier transform infrared spectroscopy (FTIR), scanning electron microscopy (SEM), and nitrogen adsorption–desorption analysis. Subsequently, the synthesized MIL-53(Al) nanoparticles were introduced as functional nanofillers into CA membranes to fabricate CA/MIL-53(Al) mixed matrix FO membranes. The effects of MIL-53(Al) loading, blending temperature, coagulation bath temperature, and pore-forming agent dosage on membrane performance were systematically investigated to optimize the fabrication parameters. The optimized membranes were further characterized using FTIR, SEM, atomic force microscopy (AFM), and water contact angle measurements to evaluate their chemical structure, surface morphology, and hydrophilicity. Finally, the separation performance of the optimized CA/MIL-53(Al) mixed matrix FO membrane was evaluated and compared with that of the pristine CA membrane.

## 2. Materials and Methods

### 2.1. Materials

CA was obtained from Jiangsu Wuxi Research and Design Institute of Chemical Industry Co., Ltd., Wuxi, Jiangsu, China. Lactic acid, N, N-dimethylformamide (DMF), N, N-dimethylacetamide (DMAc), methanol, anhydrous ethanol, sodium chloride (NaCl), and aluminum nitrate nonahydrate (Al(NO_3_)_3_·9H_2_O) were supplied by Tianjin Fuyu Fine Chemical Co., Ltd., Tianjin, China, and Tianjin Damao Chemical Reagent Factory, Tianjin, China. Formic acid and terephthalic acid (H_2_BDC) were purchased from Shanghai Macklin Biochemical Technology Co., Ltd., Shanghai, China. A non-woven polyester mesh support was provided by Shanghai Haitang Mesh Filter Fabric Weaving Co., Ltd., Shanghai, China All reagents were analytical grade and used without further purification.

### 2.2. Preparation of MOFs and Composite Membranes

#### 2.2.1. Synthesis of MIL-53(Al)

MIL-53(Al) was synthesized via a hydrothermal method according to a previously reported procedure [41]. Typically, 2.88 g of Al(NO_3_)_3_·9H_2_O was dissolved in 50 mL of N, N-dimethylformamide (DMF) under continuous magnetic stirring until a clear solution was obtained. Subsequently, 1.27 g of terephthalic acid (H_2_BDC) was added to the solution, and the mixture was stirred until the ligand was completely dissolved. The resulting precursor solution was transferred into a polytetrafluoroethylene (PTFE)-lined stainless-steel autoclave and maintained at 190 °C for 24 h in a drying oven. After naturally cooling to room temperature, the obtained solid product was collected and sequentially washed with DMF and methanol by centrifugation three times to remove residual reactants and impurities. The resulting off-white precipitate was dried and milled into a fine powder. The as-prepared MIL-53(Al) powder was stored in a sealed container under dry conditions for further characterization and membrane fabrication.

#### 2.2.2. Fabrication of CA/MIL-53(Al) Membranes

Before membrane fabrication, CA was dried in an oven at 80 °C for 24 h to remove residual moisture. A predetermined amount of MIL-53(Al) nanoparticles was dispersed in a mixed solvent system of N, N-dimethylacetamide (DMAc), lactic acid, and methanol, followed by ultrasonication to achieve uniform nanofiller dispersion. Subsequently, the obtained dispersion was transferred into a three-neck flask placed in a thermostatic oil bath, and dried CA was added. The mixture was heated and continuously stirred for 8 h at a predetermined temperature until complete dissolution of CA, resulting in a homogeneous casting solution.

The prepared casting solution was allowed to stand at room temperature for more than 20 h to eliminate entrapped air bubbles. A flat non-woven polyester mesh was placed on a smooth and dry glass plate as the supporting substrate. The degassed casting solution was uniformly spread onto the substrate using a casting knife to obtain a membrane layer with controlled thickness. After exposure to air for 30 s, the freshly cast membrane was immersed in a coagulation bath containing deionized water at a controlled temperature to induce phase inversion. After complete phase separation, the formed membrane was automatically detached from the glass plate. Finally, the as-prepared CA/MIL-53(Al) mixed matrix FO membrane was immersed in deionized water for 24 h to remove residual solvents and stored in water before further performance evaluation.

### 2.3. Material and Membrane Characterization

The crystal structure of the synthesized MIL-53(Al) samples was analyzed by X-ray diffraction (XRD, Bruker D8 Advance, Bruker AXS, Karlsruhe, Germany). The XRD measurements followed the standard operating procedure of the instrument, and the raw data were processed only with Jade(9.0) for phase identification and OriginPro(2025) for plotting; no additional smoothing or background subtraction was applied during data processing. Fourier transform infrared spectroscopy (FTIR, Nicolet iS50, Thermo Fisher Scientific, Waltham, MA, USA) was performed to identify the chemical functional groups of the samples. For FTIR, KBr pellets were used, and a background spectrum of pure KBr was recorded and subtracted before each sample measurement; the resulting spectra were plotted using Origin software without additional baseline correction or smoothing. The morphology of MIL-53(Al) and the surface/cross-sectional morphologies of the prepared membranes were examined using scanning electron microscopy (SEM, QUANTA 250 FEG, FEI, Hillsboro, OR, USA). The N_2_ adsorption–desorption isotherms of MIL-53(Al) were measured by the Brunauer–Emmett–Teller (BET) method to determine the specific surface area and pore size distribution. The surface hydrophilicity of the membranes was evaluated by water contact angle measurements at 25 °C using a contact angle goniometer (JC2000D2G, Shanghai Zhongchen Digital Technology Equipment Co., Ltd., Shanghai, China). The surface roughness of the membranes was characterized by atomic force microscopy (AFM, Bruker Dimension Icon, Bruker, Karlsruhe, Germany). Before AFM analysis, all membrane samples were thoroughly dried to remove residual moisture.

### 2.4. Performance Testing of FO Membrane Characterization

The FO performance of the fabricated membranes was evaluated based on water flux and reverse salt flux. All FO experiments were conducted in the FO mode using a laboratory-scale cross-flow system. A peristaltic pump operating at 100 rpm was used to circulate the feed solution (FS) and draw solution (DS) continuously. Deionized water and 1 mol/L NaCl aqueous solution were used as the feed and draw solutions, respectively, to establish the osmotic driving force. Both solutions were circulated in a cross-flow configuration on opposite sides of the membrane with an effective membrane area of 30 cm^2^. The feed and draw solution reservoirs were placed at the same height to minimize the influence of hydraulic pressure. During the operation, the conductivity change in the feed solution and the mass variation in the draw solution were continuously monitored using a conductivity meter and an electronic balance, respectively. Both instruments were connected to a computer for real-time data acquisition.

The water flux (*J_w_*) was calculated based on the mass increase in the draw solution according to Equation (1):

(1) $$J_{w}=\frac{∆m}{\mathrm{A\rho t}}$$

where *J_w_* represents the FO water flux (L/(m^2^·h)), Δ*m* is the mass change in the draw solution (kg), *A* is the effective membrane area (m^2^), *ρ* is the density of water (kg/m^3^), and *t* is the operating time (h). To ensure measurement accuracy and system stability, all data were collected after 5 min of continuous operation.

The reverse salt flux (*J_s_*) was determined according to the increase in NaCl concentration in the feed solution during FO operation. The salt diffusion from the draw solution to the feed solution is an undesirable phenomenon that affects membrane selectivity and long-term operation stability. The conductivity variation in the feed solution was converted into salt concentration to calculate the reverse salt flux according to Equation (2):

(2) $$J_{s}=\frac{C_{t}V_{t}-C_{0}V_{0}}{\mathrm{At}}$$

where *J_s_* is the reverse salt flux (g/(m^2^·h)), *C_t_* and *C*_0_ are the NaCl concentrations in the feed solution at time *t* and the initial stage (mol/L), respectively, *V_t_* and *V*_0_ are the corresponding feed solution volumes (L), *A* is the effective membrane area (m^2^), and *t* is the operation time (h). Similar to the water flux measurement, the RSF data were collected after 5 min of operation to ensure stable testing conditions.

## 3. Results and Discussion

### 3.1. Characterization of MIL-53(Al)

**Figure 1:**
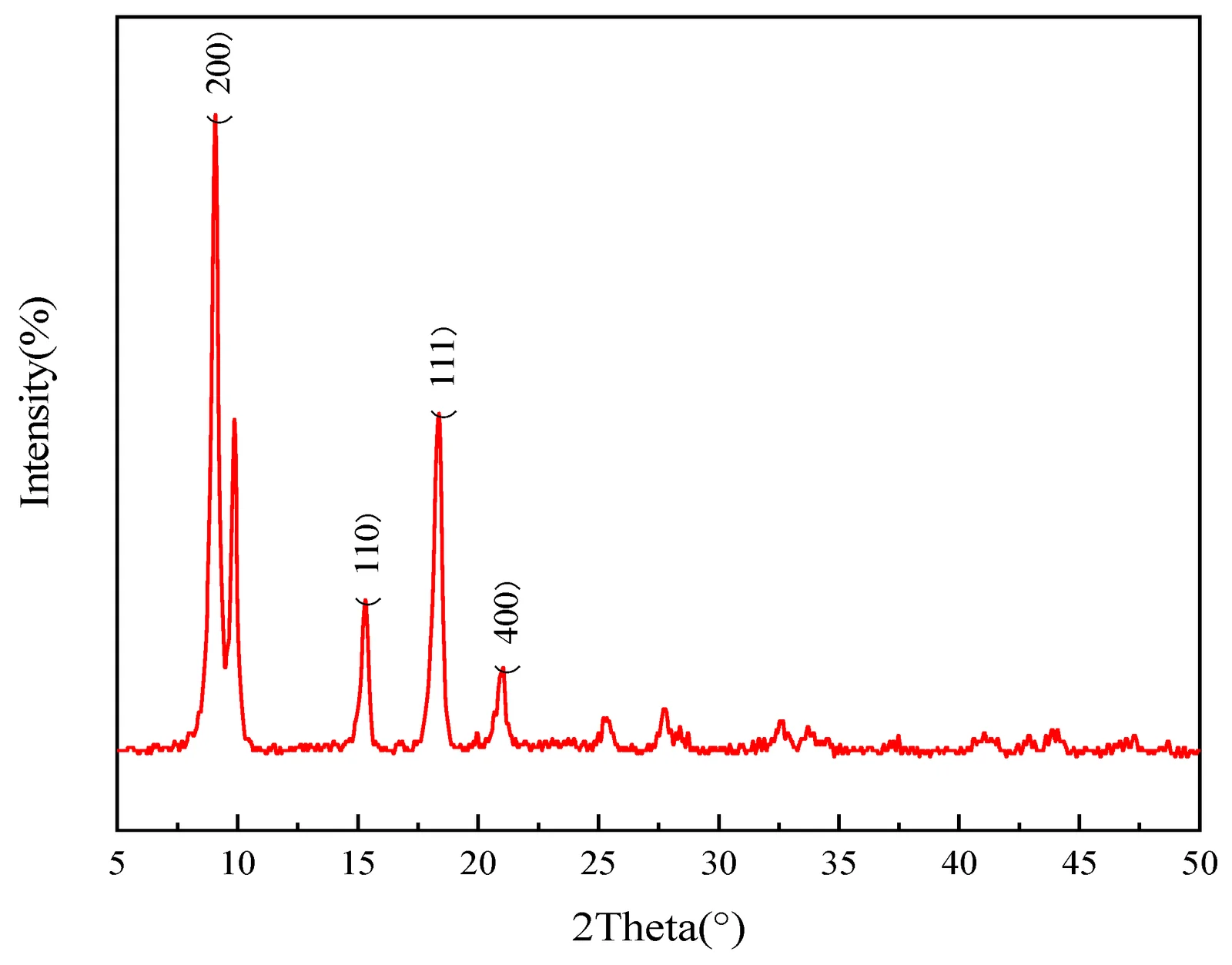
XRD pattern of MIL-53(Al).

The crystal structure of the synthesized MIL-53(Al) was analyzed by XRD, and the corresponding diffraction pattern is shown in Figure 1. The characteristic diffraction peaks of MIL-53(Al) were observed at 2θ values of 9.1°, 15.3°, 18.4°, and 21.1°, which can be assigned to the (200), (110), (111), and (400) crystal planes, respectively. The strong intensity and sharp profiles of these diffraction peaks, together with the absence of additional impurity peaks, indicate the high crystallinity and phase purity of the synthesized MIL-53(Al). Moreover, the obtained XRD pattern agrees well with the reported characteristic diffraction peaks of MIL-53(Al) in study [41], confirming the successful formation of the target MOF structure.

**Figure 2:**
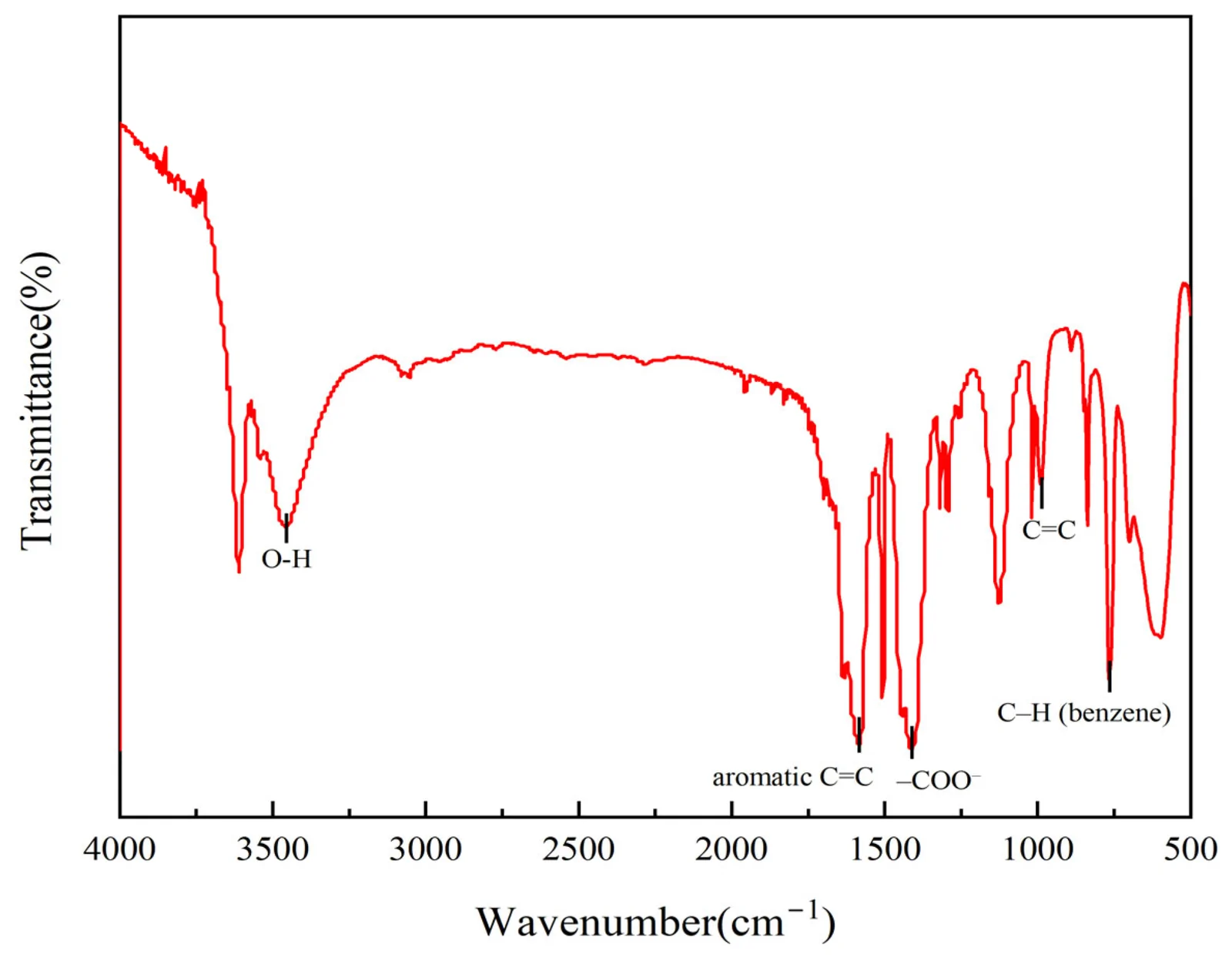
FTIR spectrum of MIL53(Al).

The chemical structure and functional groups of the synthesized MIL-53(Al) were further investigated by FTIR, and the corresponding spectrum is presented in Figure 2. The broad absorption bands located at 3450 and 3610 cm^−1^ are attributed to the O–H stretching vibrations of adsorbed water molecules. The bands appearing at 765 and 830 cm^−1^ are assigned to the out-of-plane C–H vibrations of the benzene ring, while the peak at 980 cm^−1^ corresponds to the bending vibration of =C–H groups from unsaturated hydrocarbons. The characteristic bands at 1411 and 1629 cm^−1^ are associated with the symmetric and asymmetric stretching vibrations of coordinated carboxylate groups (–COO^−^) derived from the terephthalate ligands. In addition, the peaks at 1504 and 1587 cm^−1^ are attributed to the stretching vibrations of aromatic C=C bonds. These characteristic absorption bands confirm the successful coordination between Al ions and organic ligands, demonstrating the successful synthesis of MIL-53(Al).

**Figure 3:**
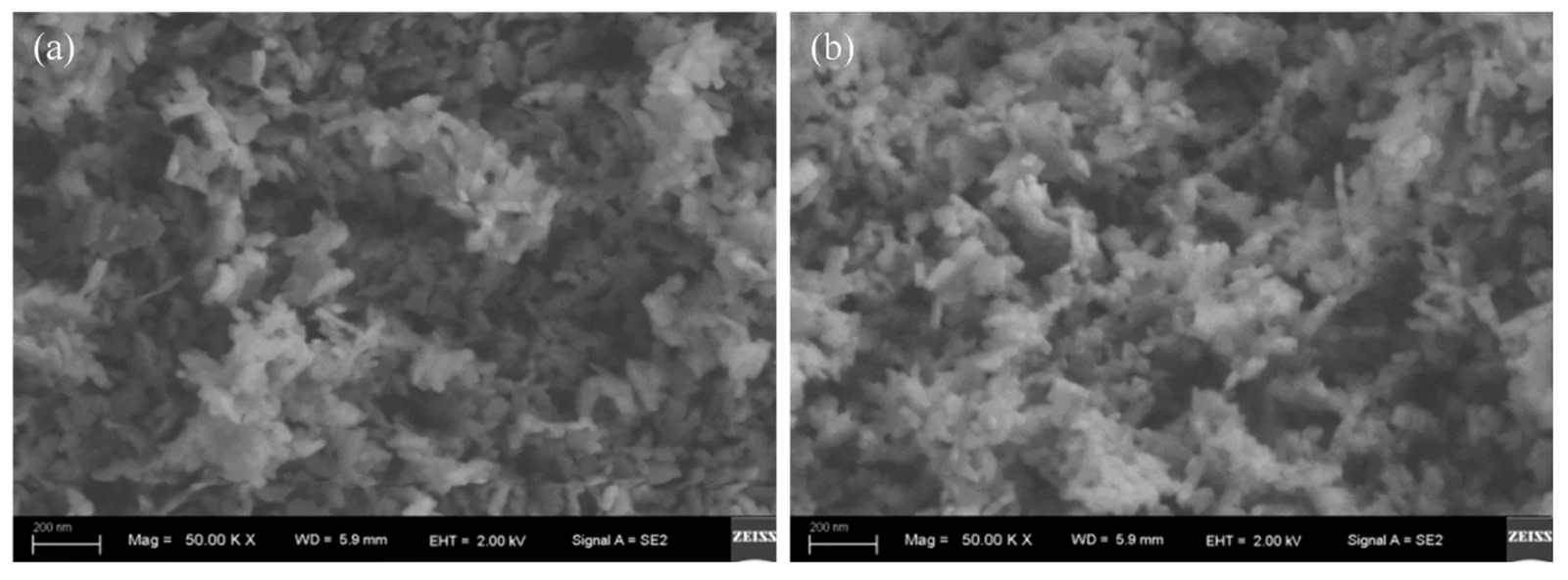
SEM image of MIL-53(Al) (**a**) SEM image of MIL-53(Al) at 50,000× magnification; (**b**) SEM image of MIL-53(Al) taken from a different region at 50,000× magnification.

The morphology of the synthesized MIL-53(Al) was examined by SEM, and the corresponding image is shown in Figure 3. As observed, MIL-53(Al) exhibits a well-defined rod-like morphology with relatively uniform particle distribution, indicating the successful formation of the MOF crystals.

**Figure 4:**
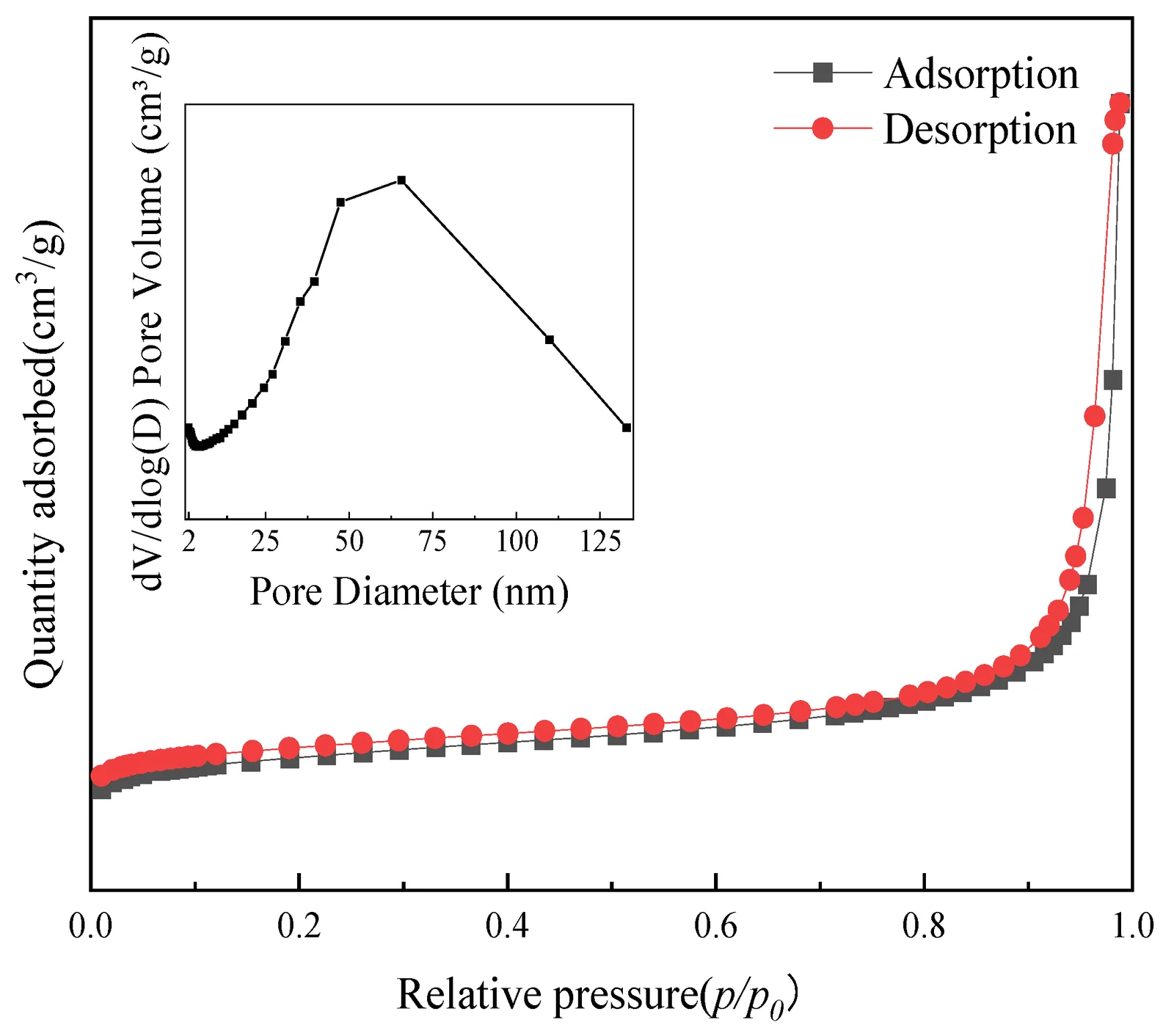
N_2_ adsorption–desorption isotherm of MIL-53(Al). Inset: pore size distribution calculated by the BJH method from the desorption branch.

The porous characteristics of MIL-53(Al) were further evaluated by N_2_ adsorption–desorption measurements, and the corresponding isotherm and pore structure parameters are presented in Figure 4 and Table 1. The obtained adsorption–desorption isotherm can be classified as a typical type IV isotherm, indicating the presence of mesoporous structures. A distinct hysteresis loop appears at a relative pressure (P/P_0_) above 0.8, which is attributed to capillary condensation occurring within the mesoporous channels. Furthermore, the pore size distribution analysis confirms that the pore structure of MIL-53(Al) is mainly located in the mesoporous range. As summarized in Table 1, the average pore diameter of MIL-53(Al) reaches 8.27 nm, further demonstrating its mesoporous characteristics. Moreover, the synthesized MIL-53(Al) possesses a considerable specific surface area of 442.55 m^2^/g and a pore volume of 0.76 cm^3^/g. The combination of abundant mesoporous channels and favorable surface area provides sufficient accessible sites for water transport and interfacial interaction, suggesting that MIL-53(Al) is a promising nanofiller for constructing high-performance mixed matrix FO membranes.

### 3.2. Optimization of MIL-53(Al) FO Membrane

To improve the permeability–selectivity performance of CA-based FO membranes, MIL-53(Al) was introduced as a functional nanofiller to construct CA/MIL-53(Al) mixed matrix FO membranes. The influences of crucial fabrication parameters, including MIL-53(Al) loading content, pore-forming agent dosage, mixing temperature, and coagulation bath temperature, on membrane structure and separation performance were systematically evaluated. The optimized preparation conditions were subsequently established to achieve enhanced water permeability and salt rejection capability.

**Figure 5:**
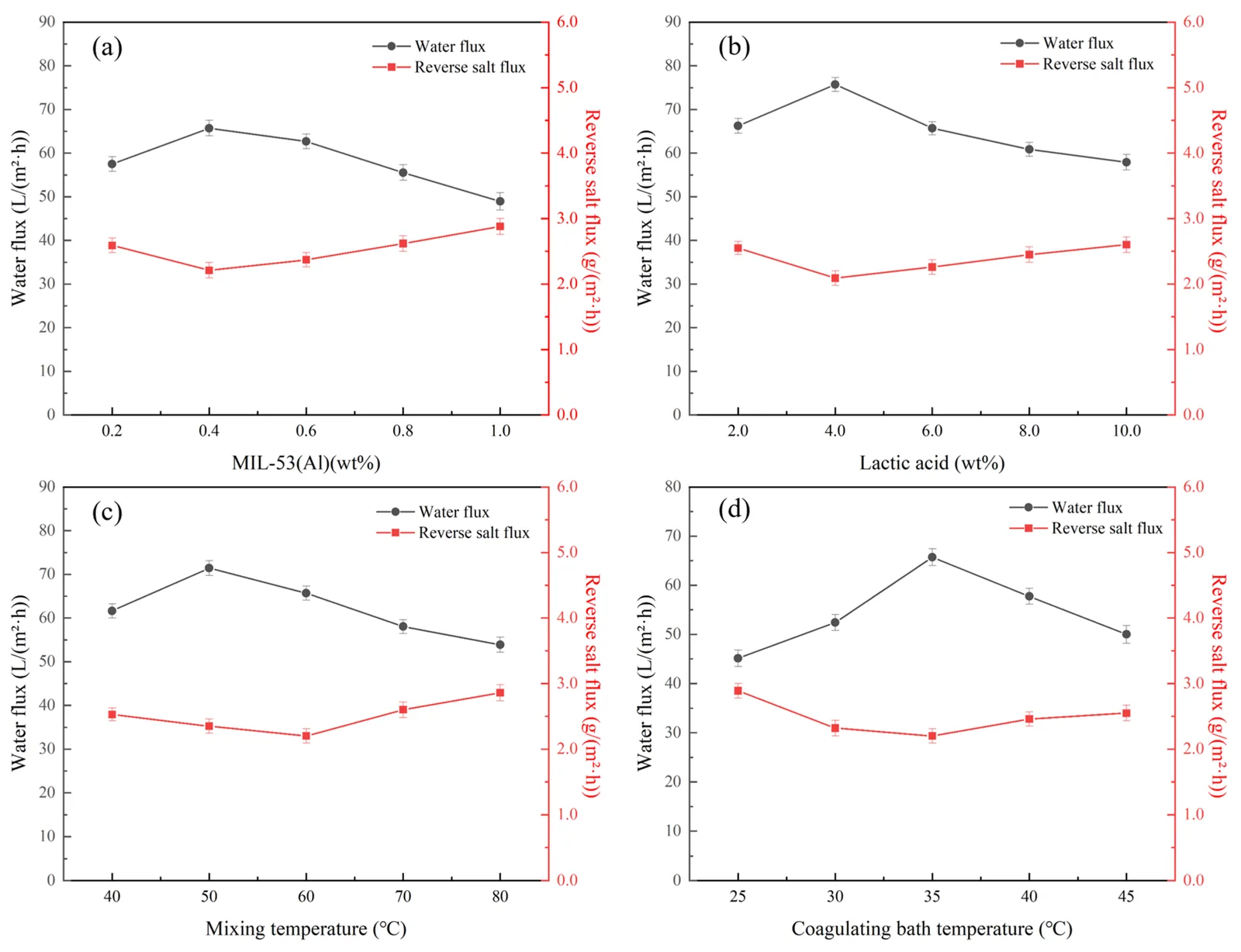
Effects of fabrication parameters on the separation performance of CA/MIL-53(Al) mixed matrix FO membranes: (**a**) MIL-53(Al) loading content; (**b**) lactic acid dosage; (**c**) mixing temperature; and (**d**) coagulation bath temperature. The other fabrication parameters were fixed as follows: (**a**) lactic acid dosage of 6.0 wt%, mixing temperature of 50 °C, and coagulation bath temperature of 30 °C; (**b**) MIL-53(Al) loading of 0.4 wt%, mixing temperature of 50 °C, and coagulation bath temperature of 30 °C; (**c**) MIL-53(Al) loading of 0.4 wt%, lactic acid dosage of 4.0 wt%, and coagulation bath temperature of 30 °C; (**d**) MIL-53(Al) loading of 0.4 wt%, lactic acid dosage of 4.0 wt%, and mixing temperature of 50 °C.

Preliminary experiments indicated that excessive incorporation of MIL-53(Al) could negatively affect membrane fabrication due to the poor dispersion stability of MIL-53(Al) nanoparticles in the organic solvent, which hindered the formation of a homogeneous casting solution. Therefore, while maintaining the CA content, mixing temperature, coagulation bath temperature, and pore-forming agent dosage unchanged, the effect of different MIL-53(Al) loading contents on the performance of CA/MIL-53(Al) mixed matrix FO membranes was systematically investigated to determine the optimal filler incorporation amount. As shown in Figure 5a, the water flux of the mixed matrix FO membranes gradually increased with increasing MIL-53(Al) loading from 0.2 to 0.4 wt%. However, a further increase in MIL-53(Al) loading beyond 0.4 wt% resulted in a decline in water flux. In contrast, the reverse salt flux remained relatively stable with increasing MIL-53(Al) content. The maximum water flux of 65.71 L/(m^2^·h) and the minimum reverse salt flux of 2.21 g/(m^2^·h) were achieved at an optimal MIL-53(Al) loading of 0.4 wt%. The enhanced water permeability can be attributed to the intrinsic hydrophilicity and porous structure of MIL-53(Al). The incorporation of hydrophilic MIL-53(Al) nanoparticles into the CA matrix improved the surface hydrophilicity of the mixed matrix membrane and provided additional water transport pathways, thereby facilitating water permeation. However, excessive addition of MIL-53(Al) likely caused nanoparticle aggregation due to insufficient dispersion in the casting solution. The aggregated particles may partially block membrane pores and increase the viscosity of the casting solution, leading to an undesirable membrane morphology and reduced water permeability. Therefore, considering the balance between water flux and reverse salt flux, 0.4 wt% was selected as the optimal MIL-53(Al) loading content for fabricating CA/MIL-53(Al) mixed matrix FO membranes.

The pore-forming agent plays an important role in determining the structure and separation performance of FO membranes. In this study, lactic acid was introduced as a pore-forming agent. While keeping the MIL-53(Al) loading, CA content, mixing temperature, and coagulation bath temperature constant, the effect of different lactic acid dosages on the performance of CA/MIL-53(Al) mixed matrix FO membranes was systematically investigated to determine the optimal pore-forming agent content. As shown in Figure 5b, with increasing lactic acid dosage, the water flux of the mixed matrix FO membranes initially increased and then decreased, whereas the reverse salt flux exhibited an opposite trend, decreasing initially and subsequently increasing. The optimal membrane performance was achieved at a lactic acid dosage of 4.0 wt%, where the membrane exhibited the highest water flux and the lowest reverse salt flux. This improvement can be attributed to the regulating effect of an appropriate amount of lactic acid on the phase inversion process. The addition of a suitable amount of lactic acid promoted the formation of an optimized selective layer and facilitated the generation of interconnected pore channels, thereby reducing water transport resistance and enhancing membrane separation performance. However, excessive lactic acid addition (>4.0 wt%) increased the viscosity of the casting solution and disturbed the phase separation process, resulting in a denser membrane surface and decreased internal porosity. Consequently, water transport was restricted, and salt diffusion was increased, leading to deteriorated membrane performance. Therefore, considering the balance between water flux and reverse salt flux, 4.0 wt% lactic acid was selected as the optimal pore-forming agent dosage for fabricating CA/MIL-53(Al) mixed matrix FO membranes.

While keeping the CA content, MIL-53(Al) loading, coagulation bath temperature, and pore-forming agent dosage constant, the influence of different mixing temperatures (40, 50, 60, 70, and 80 °C) on the performance of CA/MIL-53(Al) mixed matrix FO membranes was systematically investigated to determine the optimal fabrication temperature. As shown in Figure 5c, the water flux of the CA/MIL-53(Al) mixed matrix FO membrane initially increased and subsequently decreased with increasing mixing temperature, reaching the maximum value at 50 °C. Meanwhile, the reverse salt flux exhibited an opposite trend, decreasing initially and then increasing, with the lowest value obtained at 60 °C. The improvement in membrane performance within an appropriate temperature range can be attributed to the enhanced dissolution and dispersion of CA and MIL-53(Al) nanoparticles in the organic solvent at elevated temperatures. This resulted in a more homogeneous casting solution with reduced viscosity, which facilitated membrane formation during the phase inversion process and promoted the development of favorable membrane structures. Consequently, water transport was enhanced while reverse salt diffusion was suppressed. However, excessively high mixing temperatures adversely affected membrane formation. A visible color change in the casting solution was observed at elevated temperatures, suggesting possible changes in the polymer dissolution state and casting solution stability. The excessive thermal energy accelerated polymer chain mobility and disturbed the phase separation process, resulting in the collapse or deterioration of internal pore structures. As a consequence, water flux decreased significantly, and salt rejection performance was reduced, leading to inferior overall separation performance. Therefore, considering the balance between water permeability and salt rejection, 50 °C was selected as the optimal blending temperature for fabricating CA/MIL-53(Al) mixed matrix FO membranes.

The coagulation bath temperature plays a crucial role in regulating the solvent–non-solvent exchange rate and phase separation behavior during the phase inversion process. While keeping the MIL-53(Al) loading, CA content, mixing temperature, and pore-forming agent dosage constant, the influence of different coagulation bath temperatures (25, 30, 35, 40, and 45 °C) on the performance of CA/MIL-53(Al) mixed matrix FO membranes was systematically investigated to determine the optimal coagulation bath temperature. As shown in Figure 5d, the water flux of the mixed matrix FO membranes initially increased and subsequently decreased with increasing coagulation bath temperature, reaching the maximum value at 35 °C. Notably, the enhancement in water flux within this temperature range was significant. Meanwhile, the reverse salt flux exhibited an opposite trend, decreasing initially and then increasing, with the minimum value obtained at 35 °C. The improved membrane performance at moderate coagulation bath temperatures can be attributed to the accelerated exchange between solvent and non-solvent during phase inversion. Within the temperature range of 25–35 °C, the increased diffusion rate promoted rapid phase separation and facilitated the formation of well-developed finger-like pore structures. These interconnected porous channels effectively reduced the resistance to water transport and enhanced water permeation, resulting in increased water flux and suppressed reverse salt diffusion. However, excessively high coagulation bath temperatures negatively affected membrane formation. The accelerated polymer chain mobility altered the phase separation behavior and hindered the formation of favorable finger-like pore structures, leading to reduced membrane porosity and increased transport resistance. Consequently, the water permeability and salt rejection performance of the CA/MIL-53(Al) mixed matrix FO membranes were deteriorated. Therefore, considering the balance between water flux and reverse salt flux, 35 °C was selected as the optimal coagulation bath temperature for fabricating CA/MIL-53(Al) mixed matrix FO membranes.

### 3.3. Characterization of FO Membranes

Based on the above optimization studies, the optimal fabrication conditions for the CA/MIL-53(Al) mixed matrix FO membrane were established as follows: CA content of 12.0 wt%, MIL-53(Al) loading of 0.4 wt%, lactic acid dosage of 4.0 wt%, blending temperature of 50 °C, and coagulation bath temperature of 35 °C. Under these optimized conditions, the CA/MIL-53(Al) mixed matrix FO membrane was prepared and further investigated in comparison with the pristine CA membrane to elucidate the effects of MIL-53(Al) incorporation on membrane structure and physicochemical properties.

#### 3.3.1. FTIR Analysis

**Figure 6:**
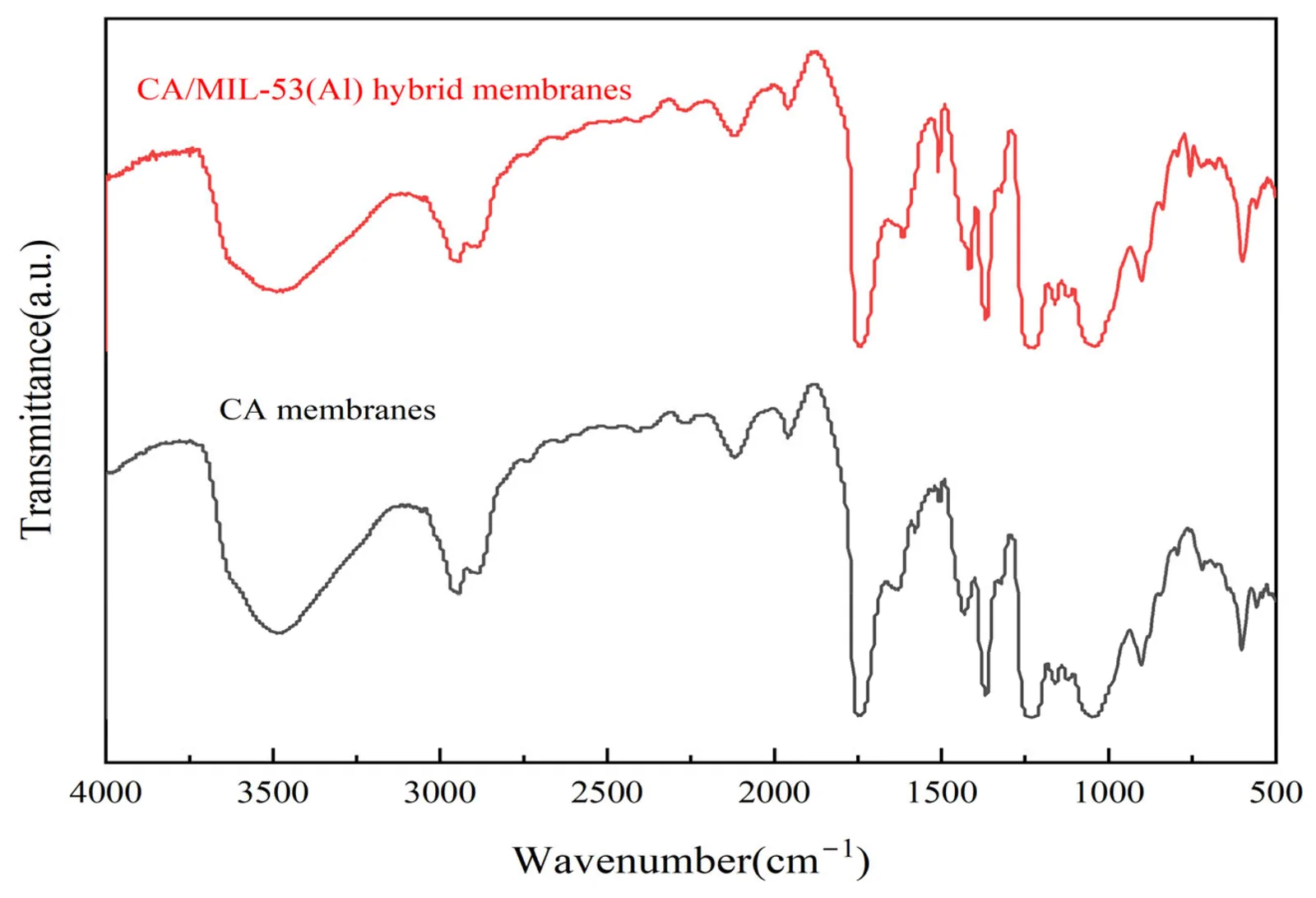
FTIR spectra of the CA membrane and the CA/MIL-53(Al) membrane.

FTIR measurements were conducted to investigate the chemical structures of the pristine CA membrane and the CA/MIL-53(Al) mixed matrix FO membrane prepared under the optimal fabrication conditions. As shown in Figure 6, the absorption band located at 1747 cm^−1^ is attributed to the stretching vibration of C=O groups, representing the characteristic signal of ester carbonyl groups in the CA matrix. The peak at 1234 cm^−1^ corresponds to the stretching vibration of C–O bonds in ester groups. The absorption bands at 1374 and 1437 cm^−1^ are assigned to the bending vibrations of C–H bonds in –CH_3_ groups, while the band at 1052 cm^−1^ is associated with the characteristic vibration of primary alcohol groups. Moreover, no obvious absorption bands were observed in the range of 1840–2887 cm^−1^, indicating the absence of residual acetic anhydride in the fabricated mixed matrix membranes. The characteristic peaks of the pristine CA membrane and the CA/MIL-53(Al) mixed matrix FO membrane appeared at nearly identical positions. This phenomenon can be attributed to the relatively low MIL-53(Al) loading content, where the characteristic signals of MIL-53(Al) were weak and partially overlapped by the strong absorption bands of the CA matrix. Therefore, both membranes exhibited similar FTIR profiles, suggesting that the incorporation of MIL-53(Al) did not alter the chemical backbone structure of CA and that MIL-53(Al) was successfully introduced into the polymer matrix without forming new chemical bonds.

#### 3.3.2. SEM Analysis

**Figure 7:**
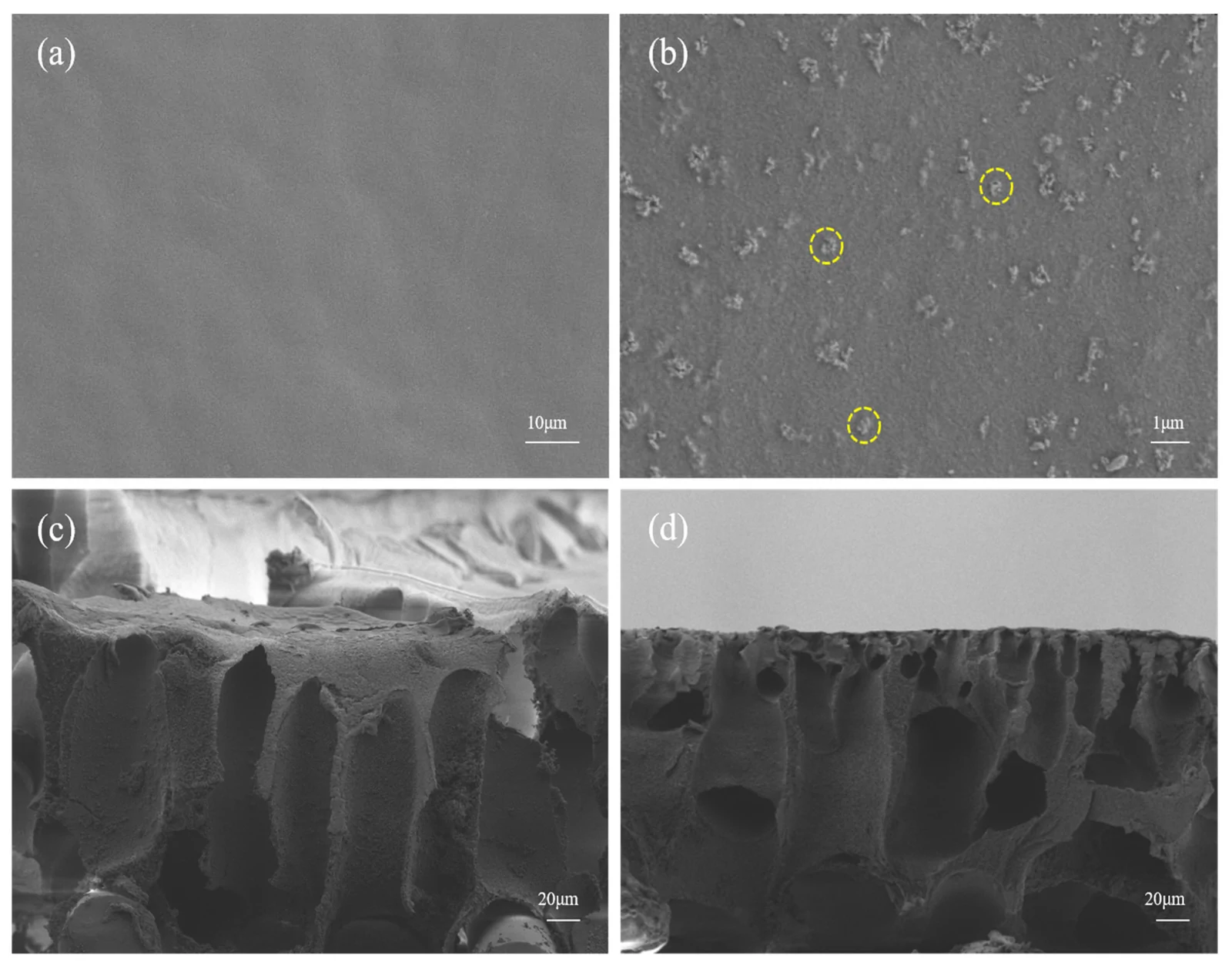
SEM images of the membranes. (**a**) Surface morphology of the CA membrane (Mag = 1.00 K X); (**b**) surface morphology of the CA/MIL-53(Al) membrane (Mag = 7.00 K X), with yellow circles indicating representative MOF particles; (**c**) cross-sectional morphology of the CA membrane (Mag = 300 X); (**d**) cross-sectional morphology of the CA/MIL-53(Al) membrane (Mag = 300 X).

SEM characterization was performed to investigate the surface and cross-sectional morphologies of the pristine CA FO membrane and the optimized CA/MIL-53(Al) mixed-matrix FO membrane. Before SEM observation, all membrane samples were sputter-coated with gold to improve electrical conductivity. As shown in Figure 7, images (a) and (b) present the surface morphologies of the pristine CA membrane and CA/MIL-53(Al) mixed matrix membrane, respectively. Compared with the pristine CA membrane, the CA/MIL-53(Al) mixed matrix membrane exhibits uniformly distributed nanoparticles on its surface, indicating the successful introduction of MIL-53(Al) nanoparticles into the CA matrix. In addition, both membranes display relatively dense surface layers, suggesting that the incorporation of MIL-53(Al) does not compromise the formation of the selective layer. The cross-sectional SEM images (c) and (d) reveal that the CA/MIL-53(Al) mixed matrix membrane possesses more developed finger-like pore structures, larger pore channels, and improved pore connectivity compared with the pristine CA membrane. The incorporation of MIL-53(Al) nanoparticles can regulate the membrane porosity and pore size distribution, thereby affecting water transport pathways and solute rejection behavior [42]. The enhanced finger-like pore structure provides reduced resistance for water transport, while the hydrophilic MIL-53(Al) nanoparticles contribute to improved water affinity, resulting in enhanced membrane permeability and separation performance. Overall, the SEM results demonstrate that MIL-53(Al) incorporation effectively modifies the internal microstructure of the CA membrane, promotes the formation of favorable porous architectures, and improves the selectivity of the CA/MIL-53(Al) mixed matrix FO membrane.

#### 3.3.3. Contact Angle Analysis

**Figure 8:**
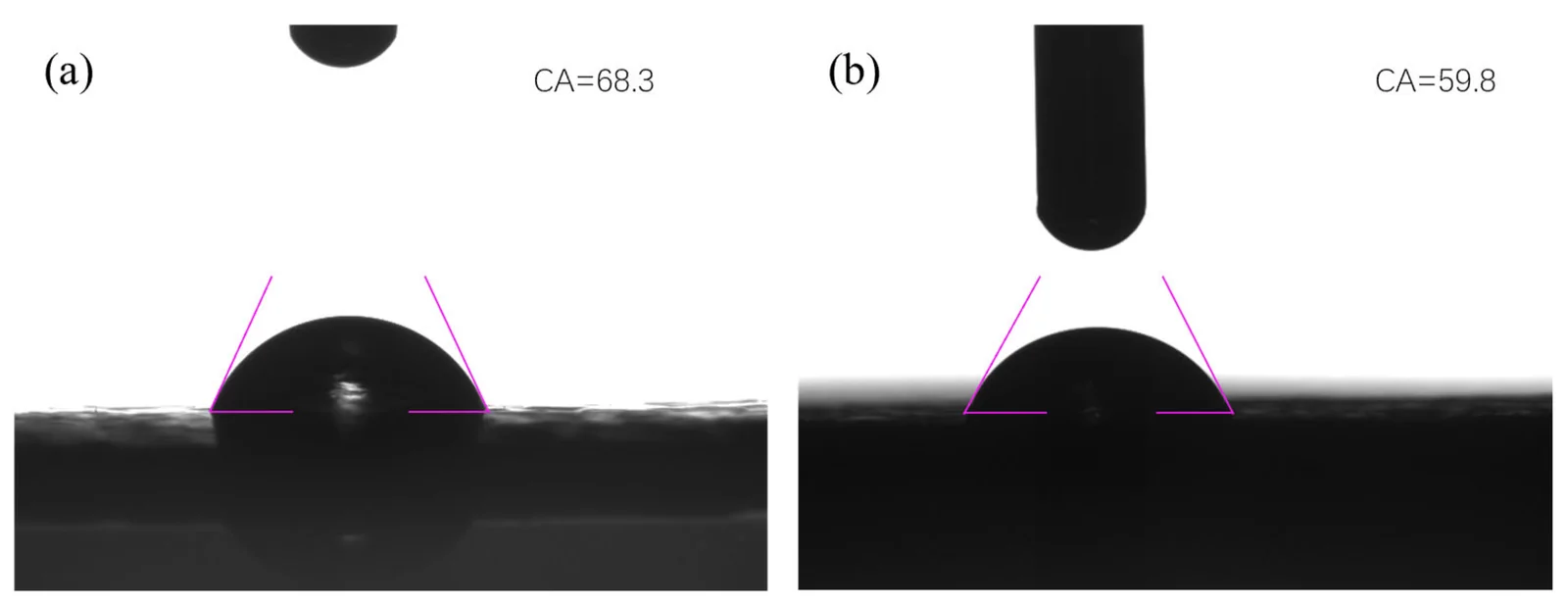
Water contact angle of the CA membrane (**a**) and the CA/MIL-53(Al) membrane (**b**). The pink lines indicate the tangent to the droplet and the baseline used to determine the contact angles of 68.3° (**a**) and 59.8° (**b**), respectively.

Water contact angle measurements were performed to evaluate the surface hydrophilicity of the pristine CA FO membrane and the optimized CA/MIL-53(Al) mixed matrix FO membrane. As shown in Figure 8, the pristine CA membrane and CA/MIL-53(Al) membrane exhibited water contact angles of 68.3° and 59.8°, respectively. Since a lower water contact angle indicates stronger surface hydrophilicity, the reduced contact angle of the CA/MIL-53(Al) membrane demonstrates its enhanced affinity toward water molecules compared with the pristine CA membrane. The improved hydrophilicity can be attributed to the incorporation of hydrophilic MIL-53(Al) nanoparticles into the CA matrix. The abundant oxygen-containing functional groups and hydrophilic surface characteristics of MIL-53(Al) facilitate water adsorption and interaction at the membrane surface, thereby improving the surface wettability. This enhanced hydrophilicity is beneficial for reducing water transport resistance and promoting water permeation through the FO membrane. Therefore, the water contact angle results confirm that MIL-53(Al) incorporation effectively modifies the surface physicochemical properties of the CA membrane and contributes to the improved separation performance of the mixed matrix FO membrane.

#### 3.3.4. AFM Analysis

**Figure 9:**
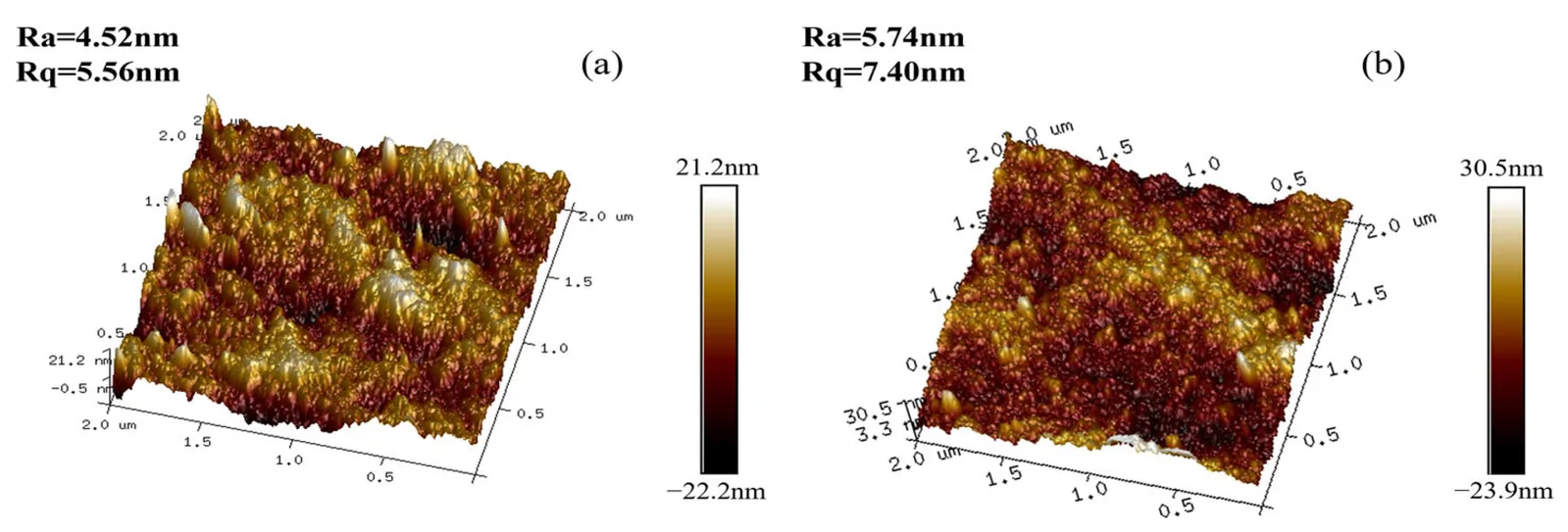
AFM images of the CA membrane (**a**) and the CA/MIL-53(Al) membrane (**b**).

AFM measurements were performed to investigate the surface topography and roughness characteristics of the pristine CA membrane and the optimized CA/MIL-53(Al) mixed matrix FO membrane. AFM is a powerful technique for obtaining nanoscale information regarding membrane surface morphology and roughness within a specific scanning area. In the AFM height images, brighter regions represent higher surface features, while roughness parameters, including average roughness (Ra) and root-mean-square roughness (Rq), are commonly used to evaluate surface irregularity and effective surface area. As shown in Figure 9, the incorporation of MIL-53(Al) nanoparticles resulted in noticeable changes in the surface morphology and roughness of the membrane. The pristine CA FO membrane exhibited Ra and Rq values of 4.52 nm and 5.56 nm, respectively. In comparison, the CA/MIL-53(Al) mixed matrix FO membrane displayed increased roughness values, with Ra and Rq reaching 5.74 nm and 7.40 nm, respectively. The increased roughness indicates that MIL-53(Al) nanoparticles effectively modified the surface structure of the CA membrane and generated a more heterogeneous surface morphology. The enhanced surface roughness can provide a larger effective surface area and more interfacial contact sites, which is beneficial for water molecule interaction and transport across the membrane. Combined with the improved hydrophilicity and optimized porous structure revealed by contact angle and SEM analyses, the increased surface roughness contributes to the enhanced permeability of the CA/MIL-53(Al) mixed matrix FO membrane. Therefore, the AFM results provide further evidence that MIL-53(Al) incorporation effectively regulates the membrane surface characteristics, contributing to the improved water flux and overall separation performance of the mixed matrix FO membrane.

### 3.4. Comparison of FO Membrane Separation Performance

**Figure 10:**
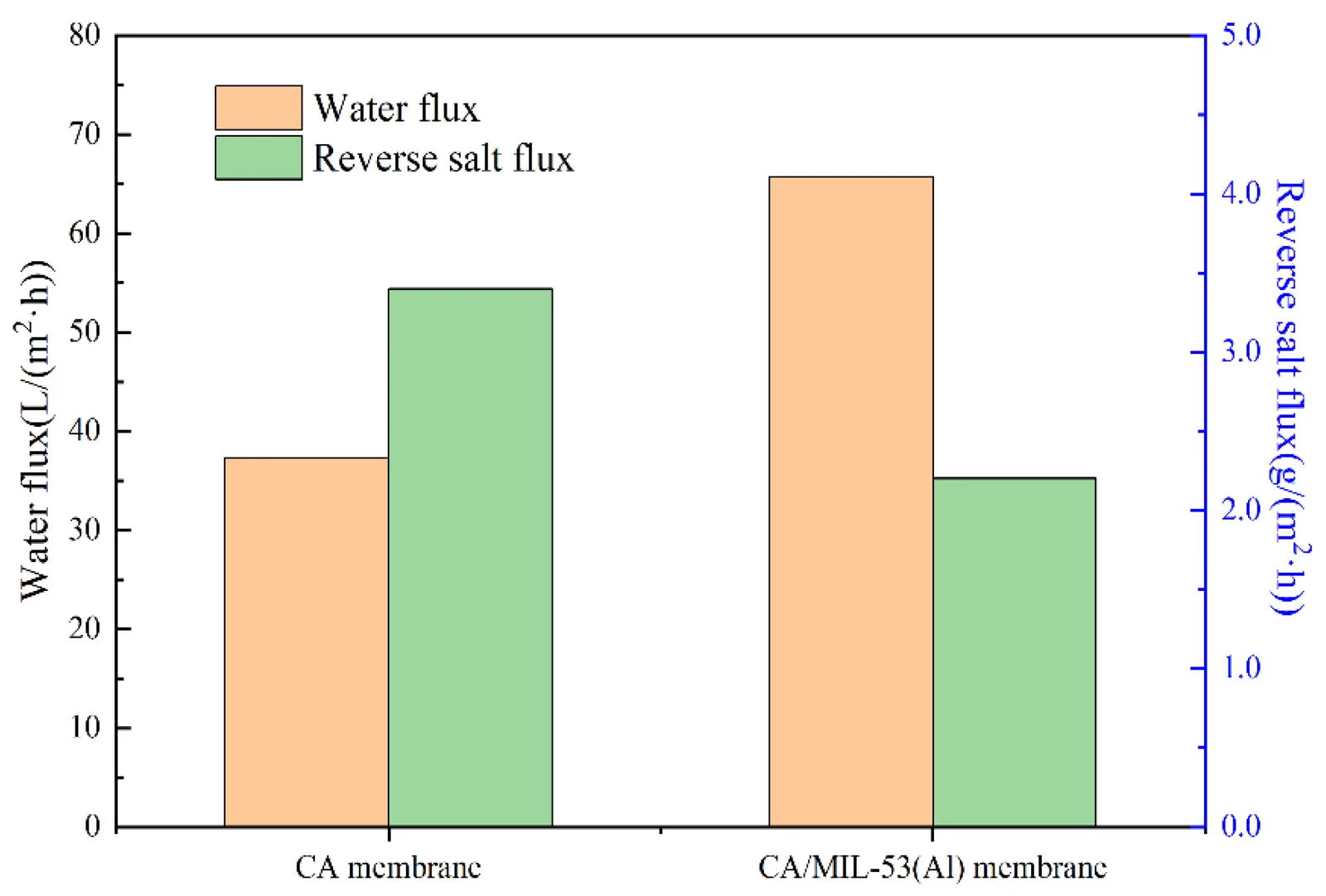
The properties of the CA membrane and the CA/MIL-53(Al) membrane.

The separation performance of the CA/MIL-53(Al) mixed matrix FO membrane fabricated under the optimal conditions was evaluated and compared with that of the pristine CA FO membrane. As shown in Figure 10, the pristine CA FO membrane exhibited a water flux of 37.26 L/(m^2^·h) and a reverse salt flux of 3.40 g/(m^2^·h). In comparison, the CA/MIL-53(Al) mixed matrix FO membrane achieved a significantly enhanced water flux of 65.71 L/(m^2^·h), accompanied by a reduced reverse salt flux of 2.20 g/(m^2^·h). These results demonstrate that the incorporation of MIL-53(Al) effectively improves both water permeability and solute rejection capability of the CA-based FO membrane. The enhanced membrane performance can be attributed to the synergistic effects induced by MIL-53(Al) nanoparticles. The introduction of MIL-53(Al) improves the surface hydrophilicity of the membrane, increases the effective surface area, and promotes the formation of favorable porous structures during the phase inversion process. These structural and physicochemical modifications facilitate water transport while suppressing salt back diffusion, thereby enhancing the overall selectivity of the CA/MIL-53(Al) mixed matrix FO membrane.

### 3.5. Seawater Desalination Performance of FO Membranes

Natural seawater served as the feed solution, and glucose solution was adopted as the draw solution to assess the seawater desalination performance of the as-prepared CA/MIL-53(Al) mixed matrix FO membrane. The permeation and desalination properties of the CA/MIL-53(Al) membrane were systematically examined and compared with those of the pristine CA membrane and commercial HTI membrane.

**Figure 11:**
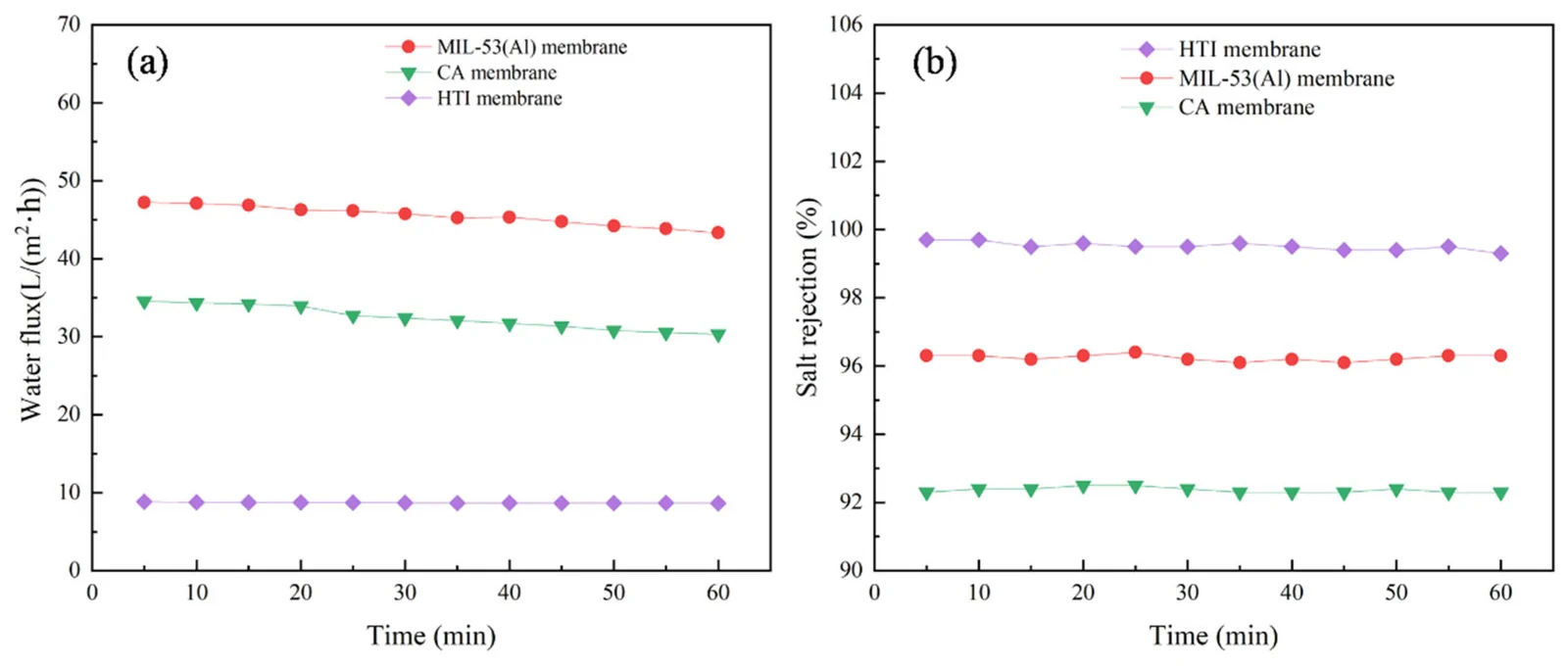
The performance of water flux (**a**) and salt rejection rate (**b**) of the FO membrane.

As shown in Figure 11a, the commercial HTI membrane exhibited a much lower water flux than the laboratory-fabricated FO membranes. Notably, the CA/MIL-53(Al) mixed matrix membrane achieved the highest water flux among all tested samples and displayed favorable operational stability during seawater desalination. As shown in Figure 11b, the CA/MIL-53(Al) mixed matrix membrane showed better salt rejection than the pristine CA membrane, though its performance was slightly inferior to that of the commercial HTI membrane. Despite the slight gap in salt rejection compared with the commercial counterpart, the CA/MIL-53(Al) membrane achieved a markedly higher water flux while maintaining satisfactory desalination efficiency. Therefore, the developed mixed matrix membrane exhibits promising preliminary potential for seawater desalination applications, although its long-term operational stability remains to be further evaluated.

## 4. Conclusions

MIL-53(Al) nanoparticles were successfully synthesized via a solvothermal route and characterized by XRD, FTIR, SEM, and N_2_ adsorption–desorption analyses. The prepared MIL-53(Al) was incorporated into CA membranes as a functional nanofiller to fabricate CA/MIL-53(Al) mixed matrix FO membranes. The effects of MIL-53(Al) loading, pore-forming agent dosage, blending temperature, and coagulation bath temperature on membrane performance were systematically investigated, and the optimal fabrication conditions were determined as follows: CA content of 12.0 wt%, MIL-53(Al) loading of 0.4 wt%, lactic acid dosage of 4.0 wt%, mixing temperature of 50 °C, and coagulation bath temperature of 35 °C. The incorporation of MIL-53(Al) effectively improved the surface hydrophilicity and roughness of the CA membrane. Compared with the pristine CA membrane, the optimized CA/MIL-53(Al) mixed matrix FO membrane exhibited a lower water contact angle (59.8°) and higher surface roughness (Ra = 5.74 nm, Rq = 7.40 nm), contributing to enhanced water transport and separation performance. The optimized membrane achieved a water flux of 65.71 L/(m^2^·h) and a reverse salt flux of 2.20 g/(m^2^·h), showing simultaneously improved permeability and salt rejection compared with the pristine CA membrane. Furthermore, seawater desalination experiments demonstrated the superior desalination performance of the CA/MIL-53(Al) membrane, indicating its promising potential for practical seawater desalination applications. This study provides an effective strategy for developing high-performance CA-based FO membranes through MOF nanofiller incorporation.

## Figures and Tables

**Table 1:** Specific surface area and pore structure parameters of MIL-53(Al).

Properties	Values
BET surface area (m^2^/g)	442.55
Langmuir surface area (m^2^/g)	715.16
Micropore surface area (m^2^/g)	303.51
Pore volume (cm^3^/g)	0.76
Average pore size (nm)	8.27

## Data Availability

The data presented in this study are available from the corresponding authors upon reasonable request.
